# Bias of the additive hazard model in the presence of causal effect heterogeneity

**DOI:** 10.1007/s10985-024-09616-z

**Published:** 2024-03-11

**Authors:** Richard A. J. Post, Edwin R. van den Heuvel, Hein Putter

**Affiliations:** 1 Department of Mathematics and Computer Science, Eindhoven University of Technology, Eindhoven, The Netherlands; 2 Department of Biomedical Data Sciences, Leiden University Medical Center, Leiden, The Netherlands; 3 Mathematical Institute, Leiden University, Leiden, The Netherlands

**Keywords:** Causal inference, Causal hazard difference, Individual effect modification, Selection bias, Aalen additive hazard model, 62D20, 62N02

## Abstract

Hazard ratios are prone to selection bias, compromising their use as causal estimands. On the other hand, if Aalen’s additive hazard model applies, the hazard difference has been shown to remain unaffected by the selection of frailty factors over time. Then, in the absence of confounding, observed hazard differences are equal in expectation to the causal hazard differences. However, in the presence of effect (on the hazard) heterogeneity, the observed hazard difference is also affected by selection of survivors. In this work, we formalize how the observed hazard difference (from a randomized controlled trial) evolves by selecting favourable levels of effect modifiers in the exposed group and thus deviates from the causal effect of interest. Such selection may result in a non-linear integrated hazard difference curve even when the individual causal effects are time-invariant. Therefore, a homogeneous time-varying causal additive effect on the hazard cannot be distinguished from a time-invariant but heterogeneous causal effect. We illustrate this causal issue by studying the effect of chemotherapy on the survival time of patients suffering from carcinoma of the oropharynx using data from a clinical trial. The hazard difference can thus not be used as an appropriate measure of the causal effect without making untestable assumptions.

## Introduction

Hazard ratios, often obtained by fitting a Cox proportional hazards model (Cox 1972), are the most common effect measures when dealing with time-to-event data. However, the hazard ratio is prone to selection bias due to conditioning on survival and therefore not suitable for causal inference (Hernán 2010; Aalen et al. 2015; Stensrud et al. 2017). It has been recommended to use other, better interpretable, estimands when interested in causal effects (Hernán 2010; Stensrud et al. 2018; Bartlett et al. 2020; Young et al. 2020). Alternatively, using the additive hazard model can avoid interpretation issues (Aalen et al. 2015; Martinussen et al. 2020). In the nonparametric model proposed by Aalen (1989), the hazard rate at time *t* for individual *i* with (possibly time-dependent) covariates $\varvec{x}_{i}(t)$ (of dimension *p*) is determined by the values of $\varvec{x}_{i}$ up until time *t* and equals

$$\begin{aligned} \lambda \left( t \mid \{ \varvec{x}_{i}(s) \}_{s\le t}\right) = \beta _{0}(t) + \beta _{1}(t) x_{i1}(t) + \ldots + \beta _{p}(t) x_{ip}(t), \end{aligned}$$

where the parameters $\beta _{j}(t)$ are arbitrary regression functions, allowing time-varying effects (Aalen et al. 2008). Restricted versions have been proposed by Lin and Ying (1994) and McKeague and Sasieni (1994), where all or some $\beta _{j}(t)$ are assumed to be constant over time. The cumulative regression function, $B_{j}(t) = \int _{0}^{t} \beta _{j}(s) ds$, may reveal changes in effect over time, see for example Aalen et al. (2008, pp. 160-162).

For cause-effect relations that can be accurately described with Aalen’s additive hazard model, the hazard difference is a collapsible measure (Martinussen and Vansteelandt 2013). Then, in the absence of confounding, the hazard difference can be appropriately used to estimate the causal effect, even in the case of unmeasured risk factors (Aalen et al. 2015). For this, it is necessary that the exposure effect on the hazard does not depend on unmeasured individual features (modifiers) and thus is the same for all individuals. However, due to the fundamental problem of causal inference, the effect homogeneity assumption is untestable (Holland 1986). In our companion paper (Post et al. 2024), we showed that next to unmeasured risk factors, i.e. frailty (Aalen et al. 2008, Chapter 6), effect heterogeneity at the level of the individual hazard results in selection bias of observed hazard ratios.

In this work, we extend the additive hazard model studied in Aalen et al. (2015) by allowing heterogeneity of the effect (on the hazard) and quantify the bias of using the observed hazard difference when estimating the causal effect. In Sect. 2, we introduce notation to describe the cause-effect relations using a structural causal model for which we can define the causal hazard difference. In practice, when appyling an additive hazard model the observed hazard difference is modelled. We show that (in absence of confounding) the expected value of the observed hazard equals a causal hazard marginalized over survivors. The expectation of the observed hazard difference thus equals a difference between hazards marginalized over survivors in the exposed and unexposed universe. By selection of individuals with favourable values of the effect modifier, this difference can deviate from the causal hazard difference as we formalize in Sect. 3. We present numerical examples to illustrate how this selection can result in a non-linear integrated hazard difference curve, that can be interpeted as reflecting a time-varying causal effect, while the actual individual causal effects are time-invariant. To emphasize why it is important to be aware of such a difference between the expected observed hazard difference and the causal hazard difference, we reflect on the analysis of the effect of treatment on survival with carcinoma of the oropharynx from the clinical trial in Sect. 4. Finally, we present some concluding remarks in Sect. 5.

## Notation and hazard differences

Probability distributions of factual and counterfactual outcomes are defined in the potential outcome framework (Neyman 1923; Rubin 1974). Let $$T_{i}$$ and $$A_{i}$$ represent the (factual) stochastic outcome and exposure assignment level of individual *i*. Let $$T_{i}^{a}$$ equal the potential outcome of individual *i* under the intervention of the exposure to level *a* (counterfactual when $$A_{i}~{\ne }~a$$). For those more familiar with the do-calculus, $$T^{a}$$ is equivalent to $$T \mid do(A{=}a)$$ as e.g. derived in Pearl (2009a, Equation 40) and Bongers et al. (2021, Definition 8.6). Throughout this paper, we will assume causal consistency, i.e. if $$A_{i}{=}a$$, then $$T_{i}^{a} = T_{i}^{A_{i}} = T_{i}$$. Causal consistency implies that potential outcomes are independent of the assigned exposure levels of other individuals. The hazard rate of the potential outcome can vary among individuals due to heterogeneity in risk factors $$U_{0}$$ as also considered by Aalen et al. (2015). The hazard difference of the potential outcomes with and without ($$a = 0)$$ exposure might also vary among individuals due to an effect modifier $$U_{1}$$. Therefore, the hazard rate of individual *i* at time *t* of the potential outcome under exposure to level *a* is a function of $$U_{0i}$$ and $$U_{1i}$$ and thus random and equals

1 $$\begin{aligned} \lambda _{i}^{a}(t) = \lim _{h\rightarrow 0}h^{-1}\mathbb {P}\left( T_{i}^{a} ~{\in }~[t,t+h) \mid T_{i}^{a}{\ge }t, U_{0i}, U_{1i}\right) . \end{aligned}$$

The hazard of the potential outcome $T_{i}^{a}$ can be parameterized with a function that depends on $U_{0i}$, $U_{1i}$ and *a*.

We describe cause-effect relations with a structural causal model (SCM) which is commonly used in the causal graphical literature, see e.g. Pearl (2009b, Chapter 1.4) and  Peters et al. (2018, Chapter 6), to model observations. Instead, we include details on individual effect modifier $U_{1}$ as well as the latent common cause of the outcomes $U_{0}$, so that the SCM consists of a joint probability distribution of $(N_{A}, U_{0}, U_{1}, N_{T})$ and a collection of structural assignments (for more details, see Post et al. (2024, Section 2)) such that 
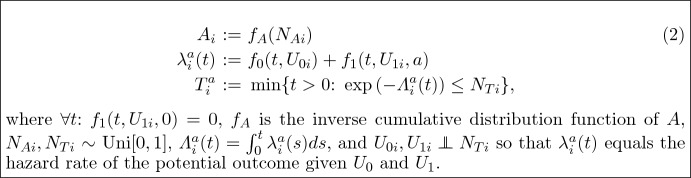


If in SCM (2), , then there exists confounding as the distributions of $(U_{0}, U_{1}, N_{T})$ are not exchangeable between exposed and non-exposed individuals. However, in this work we focus on the distribution of data observed from a properly executed RCT, where by the randomization $N_{A} {\mathrel{\perp\!\!\!\perp}} U_{0}, U_{1}, N_{T}$ so that there is no confounding. It is important to realize that a SCM cannot be validated with data as it describes potential outcomes from different universes. For each individual the outcome can only be observed in one of the universes, and only the fit of the distribution of the outcomes in the factual world can be verified. In SCM (2), we did not restrict the distribution of $U_{0}$ and $U_{1}$ and only restricted $f_{\lambda }$ and $f_{A}$ to be properly defined hazard and inverse cumulative distribution functions respectively, so that the structural model is quite general. However, in this work we limit ourselves to cause-effect relations where the causal effect of the exposure is described by $\lambda _{i}^{a}(t)-\lambda _{i}^{0}(t) = f_{1}(t,U_{1i},a)$.

### Hazard differences

If SCM (2) applies and $\lambda _{i}^{a}(t)-\lambda _{i}^{0}(t) = f_{1}(t, a)$, then the causal effect is equal for each individual, i.e. effect homogeneity, and Aalen’s additive hazard model applies. Otherwise, when $\lambda _{i}^{a}(t)-\lambda _{i}^{0}(t)=f_{1}(t, U_{1i}, a)$, the difference differs among individuals so that $\mathbb {E}[\lambda _{i}^{a}(t)-\lambda _{i}^{0}(t)]$ will typically be the estimand of interest. The latter contrast equals the difference between the expected hazard rate in the world where everyone is exposed to *a* and the world without exposure, and will therefore be referred to as the causal hazard difference (CHD) defined in Definition 1.

#### Definition 1

**Causal hazard difference** The CHD for cause-effect relations that can be parameterized with SCM 2 equals

$$\begin{aligned}\mathbb {E}&\left[ \lambda _{i}^{a}(t)\right] -\mathbb {E}\left[ \lambda _{i}^{0}(t)\right] = \mathbb {E}[f_{1}(t,U_{1},a)] \\ =&\int \lim _{h\rightarrow 0}h^{-1}\mathbb {P}\left( T^{a} ~{\in }~[t,t+h) \mid T^{a}{\ge }t, U_{0}, U_{1} \right) dF_{U_{0}, U_{1}}\\ &-\int \lim _{h\rightarrow 0}h^{-1}\mathbb {P}\left( T^{0} ~{\in }~[t,t+h) \mid T^{0}{\ge } t, U_{0} \right) dF_{U_{0}}. \end{aligned}$$

Throughout this paper, we abbreviate the Lebesque-Stieltjes integral of a function *g* with respect to probability law $F_{X}$, $\int g(x) dF_{X}(x)$, as $\int g(X) dF_{X}$.

The CHD thus equals the difference of hazard rates of the potential outcomes marginalized over the population distribution of $(U_{0}, U_{1})$. However, the distribution of $(U_{0}, U_{1})$ among survivors will differ over time (in all worlds), i.e. $(U_{0},U_{1}) \overset{d}{\ne }\ (U_{0},U_{1}) \mid T^{a}{\ge }t$. In turn, $(U_{0},U_{1}) \mid T^{a}{\ge }t$ and $(U_{0},U_{1}) \mid T^{0}{\ge }t$ can differ in distribution. As a consequence, the hazard rates for the observed (factual) outcomes are affected by these conditional distributions of $U_{0}$ and $U_{1}$, and the observed hazard difference may reflect both the causal effect of exposure and a difference in distributions of $(U_{0}, U_{1})$ between exposed and unexposed individuals. Since practioners are typically interested in the causal effect alone, it is important to understand this mixture. Since the focus of this work is on estimands, for readability, we refer to the expected value of the difference of the observed hazards as the observed hazard difference (OHD) presented in Definition 2.

#### Definition 2

**Observed hazard difference** The OHD at time *t* equals

$$\begin{aligned}&\lim _{h\rightarrow 0}h^{-1}\mathbb {P}\left( T ~{\in }~[t,t+h) \mid T{\ge }t, A{=}a \right) -\lim _{h\rightarrow 0}h^{-1}\mathbb {P}\left( T ~{\in }~[t,t+h) \mid T{\ge }t, A{=}0 \right) \\ =&\lim _{h\rightarrow 0}\int h^{-1}\mathbb {P}\left( T ~{\in }~[t,t+h) \mid T{\ge }t, A{=}a, U_{0}, U_{1} \right) dF_{U_{0}, U_{1}{\mid }T{\ge }t, A{=}a}\\ {}&-\lim _{h\rightarrow 0} \int h^{-1}\mathbb {P}\left( T ~{\in }~[t,t+h) \mid T{\ge }t, A{=}0, U_{0} \right) dF_{U_{0}{\mid }T{\ge }t, A{=}0}. \end{aligned}$$

To be precise, at time *t* the hazard rate can only be observed for non-censored individuals at that time $(C(t)=0)$. However, in this work we will assume independent censoring, so that $\mathbb {P}\left( T \mid T{\ge }t, A{=}a \right)$ is equal to $\mathbb {P}\left( T \mid T{\ge }t, A{=}a, C(t){=}0\right)$.

To compare the OHD to the CHD of interest, the OHD should be expressed in terms of potential outcomes. By causal consistency,

3 $$\begin{aligned} \mathbb {P}\left( T ~{\in }~[t,t+h) \mid T{\ge }t, A{=}a \right) = \mathbb {P}\left( T^{a} ~{\in }~[t,t+h) \mid T^{a}{\ge }t, A{=}a \right) . \end{aligned}$$

For a randomized controlled trial (RCT), where by design of the trial $A {\mathrel{\perp\!\!\!\perp}} T^{a}$ (in SCM (2) equivalent to $N_{A} {\mathrel{\perp\!\!\!\perp}} U_{0}, U_{1},N_{T}$,

4 $$\begin{aligned} \mathbb {P}\left( T^{a} ~{\in }~[t,t+h) \mid T^{a}{\ge }t, A{=}a \right) = \mathbb {P}\left( T^{a} ~{\in }~[t,t+h) \mid T^{a}{\ge }t \right) . \end{aligned}$$

The OHD at time *t* is then equal to

5 $$\begin{aligned} \lim _{h\rightarrow 0}h^{-1} \mathbb {P}\left( T^{a} ~{\in }~[t,t+h) \mid T^{a}{\ge }t \right) - \lim _{h\rightarrow 0}h^{-1}\mathbb {P}\left( T^{0} ~{\in }~[t,t+h) \mid T^{0}{\ge }t \right) . \end{aligned}$$

We refer to (5) as the survivor marginalized causal hazard difference (SMCHD) that is rewritten in Definition 3.

#### Definition 3

**Survivor marginalized causal hazard difference** The SMCHD at time *t* for cause-effect relations that can be parameterized with SCM 2 equals

$$\begin{aligned}&\lim _{h\rightarrow 0}\int h^{-1}\mathbb {P}\left( T^{a} ~{\in }~[t,t+h) {\mid }T^{a}{\ge }t, U_{0}, U_{1} \right) dF_{U_{0}, U_{1}{\mid }T^{a}{\ge }t}\\&\qquad - \int h^{-1}\mathbb {P}\left( T^{0} ~{\in }~[t,t+h) {\mid } T^{0}{\ge } t, U_{0} \right) dF_{U_{0}{\mid }T^{0}{\ge }t}. \end{aligned}$$

As the integration in Definition 1 is with respect to the population distribution of $(U_{0}, U_{1})$ (instead of that of the survivors in the exposed and unexposed universe respectively), the SMCHD is thus affected by the difference in distribution between $(U_{0}, U_{1})$ and $(U_{0}, U_{1}) \mid T^{a}\ge t$ as well as the actual causal effect. Therefore, the SMCHD can deviate from the CHD. Nevertheless, Aalen et al. (2015) explained that $U_{0} {\mathrel{\perp\!\!\!\perp}} A | T \ge t$ so that for degenerate $U_{1}$, the SMCHD is only affected by the causal effect and equals the CHD, so that the latter can be unbiasedly estimated from RCT data. In the next section, we formalize the SMCHD in case of effect heterogeneity (non-degenerate $U_{1}$) and show that , so that then the SMCHD deviates from the CHD.

## Results

In the remainder of the paper, we will focus on binary exposures such that $a ~{\in }~\{0, 1\}$. In this section we quantify how the SMCHD describes both the causal effect and the difference in distribution of $(U_{0}, U_{1})$ between survivors in the exposed and unexposed universes.

For cause-effect relations where SCM (2) applies with $\lambda _{i}^{a}(t) = f_{0}(t,U_{0i})+f_{1}(t,a)$, it is known from Aalen et al. (2015) that for an RCT $U_{0i} {\mathrel{\perp\!\!\!\perp}} A_{i} | T_{i} \ge t$, so that $U_{0}$ remains exchangeable between exposed ($U_{0} \mid T{\ge }t, A{=}1$) and nonexposed ($U_{0} \mid T{\ge }t,A{=}0$) survivors. This independence, causal consistency, and the absence of confounding in an RCT $(T_{a} {\mathrel{\perp\!\!\!\perp}} A)$ imply

$$\begin{aligned} U_{0} \mid T{\ge }t&~\overset{d}{=}~ U_{0} \mid T{\ge }t, A{=}a\\&~\overset{d}{=}~ U_{0} \mid T^{a}{\ge }t, A{=}a\\ {}&~\overset{d}{=}~ U_{0} \mid T^{a}{\ge }t. \end{aligned}$$

Thus in absence of effect heterogeneity of the hazard difference, $U_{0}$ is exchangeable between survivors in the exposed ($U_{0} \mid T^{1}{\ge }t$) and unexposed universes $(U_{0} \mid T^{0}{\ge }t)$, so that the OHD from an RCT (that equals the SMCHD) equals the CHD and describes the causal effect.

However, if heterogeneity exists, there will also be a selection of the modifier ($U_{1}$) in the exposed universe, where individuals with more favourable levels of $U_{1}$ are more likely to survive. As a result of this selection, the SMCHD over time no longer represents the (population) average effect. For the main result of this paper, we consider hazard functions that satisfy Condition 1.

### Condition 1

**Hazard without infinite discontinuity**

$$\begin{aligned} \forall t{>}0{:}~\exists \tilde{{}h}{>}0 \text { such that }\forall h^{*} ~{\in }~(0,\tilde{{}h}){:}~ \mathbb {E}\left[ f_{0}(t+h^{*},U_{0})+f_{1}(t+h^{*},U_{1},a) \mid T^{a}{\ge } t\right] <\infty \end{aligned}$$

In Theorem 1, we show that the SMCHD, for a hazard function that satisfies Condition 1, can be expressed in terms of conditional expectations of $f_{1}(t, U_{1}, 1)$ and $f_{0}(t,U_{0})$. In presence of effect heterogeneity, the SMCHD thus deviates from the CHD equal to $\mathbb {E}[f_{1}(t,U_{1},1)]$.

### Theorem 1

If the cause-effect relations of interest can be parameterized with SCM (2), where

$$\begin{aligned}\lambda _{i}^{a}(t)~{:=}~ f_{0}(t,U_{0i})+f_{1}(t,U_{1i},a), \end{aligned}$$

and Condition 1 applies, then the SMCHD at time *t* equals

$$\begin{aligned} \mathbb {E}[ f_{1}(t,U_{1},1) \mid T^{1}{\ge }t] + \mathbb {E}[ f_{0}(t,U_{0}) \mid T^{1}{\ge }t] -\mathbb {E}[f_{0}(t,U_{0}) \mid T^{0}{\ge }t]. \end{aligned}$$

To illustrate how the SMCHD can deviate from the CHD we continue by presenting some examples and apply Theorem 1. All programming codes used for these examples can be found online at https://github.com/RAJP93/CHD. First, we consider cause-effect relations for which $U_{0} {\mathrel{\perp\!\!\!\perp}} U_{1}$.

### Independent $U_{0}$ and $U_{1}$

As discussed at the start of this section, Aalen et al. (2015) implicitly showed that $U_{0} \mid T^{1}{\ge }t \overset{d}{=} U_{0} \mid T^{0}{\ge }t$ in absence of effect heterogeneity of the hazard difference. Based on similar arguments, Lemma 1 states that the additive frailty is also exchangeable in the presence of effect heterogeneity at the hazard scale that is independent of the frailty.

#### Lemma 1

If the cause-effect relations of interest can be parameterized with SCM (2), where

$$\begin{aligned}\lambda _{i}^{a}(t)~{:=}~ f_{0}(t,U_{0i})+f_{1}(t,U_{1i},a), \end{aligned}$$

and $$U_{0i} {\mathrel{\perp\!\!\!\perp}} U_{1i}$$ then,

$$\begin{aligned} \mathbb {E}\left[ f_{0}(t,U_{0}) \mid T^{1}{\ge }t\right] = \mathbb {E}\left[ f_{0}(t,U_{0}) \mid T^{0}{\ge }t\right] . \end{aligned}$$

Note that while $\mathbb {E}\left[ f_{0}(t, U_{0}) \mid T^{1}{\ge }t\right] = \mathbb {E}\left[ f_{0}(t, U_{0}) \mid T^{0}{\ge }t\right]$, $\mathbb {E}\left[ f_{0}(t, U_{0}) \mid T^{a}{\ge }t\right] \ne \mathbb {E}\left[ f_{0}(t, U_{0})\right]$ as the conditional expectations will decrease over time representing the survival of less susceptible individuals. If $U_{0} {\mathrel{\perp\!\!\!\perp}} U_{1}$, as for the case of effect homogeneity, $U_{0}$ is thus exchangeable between survivors in the exposed ($U_{0} \mid T^{1}{\ge }t$) and unexposed ($U_{0} \mid T^{0}{\ge }t$) universes. By Theorem 1 and Lemma 1, the SMCHD at time *t* now equals $\mathbb {E}[ f_{1}(t,U_{1},1) \mid T^{1}{\ge }t]$.

Let us consider cause-effect relations for which SCM (2) applies with

6 $$\begin{aligned} f_{1}(t,U_{1i},a)=U_{1i}f_{1}(t,a), \end{aligned}$$

where $f_{1}(t,0){=}~0$, then $\mathbb {E}\left[ f_{1}(t,U_{1},1) \mid T^{1}{\ge }t\right] = f_{1}(t,a) \mathbb {E}[U_{1} \mid T^{1}{\ge }t]$. By Definition 1, the CHD equals $f_{1}(t,a)\mathbb {E}[U_{1}]$, so that the difference with the SMCHD varies over time and equals

7 $$\begin{aligned} f_{1}(t,a)\left( \mathbb {E}[U_{1}]-\mathbb {E}[U_{1} \mid T^{1}{\ge }t]\right) . \end{aligned}$$

For this multiplicative case, the conditional expectation $\mathbb {E}[U_{1} \mid T^{1}{\ge }t]$ can be expressed in terms of the Laplace transform of $U_{1}$, as stated in Lemma 2.

#### Lemma 2

If the cause-effect relations of interest can be parameterized with SCM (2), where

$$\begin{aligned}f_{1}(t,U_{1i},1) = U_{1i}f_{1}(t,1), \end{aligned}$$

and $$U_{0i} {\mathrel{\perp\!\!\!\perp}} U_{1i}$$, then

$$\begin{aligned} \mathbb {E}\left[ U_{1} \mid T^{1}{\ge }t\right] = -\frac{\mathcal {L}_{U_{1}}^{'}(\int _{0}^{t}f_{1}(s,1)ds)}{\mathcal {L}_{U_{1}}(\int _{0}^{t}f_{1}(s,1)ds)}, \end{aligned}$$

where $\mathcal {L}_{U_{1}}(c) = \mathbb {E}\left[ \exp \left( -cU_{1}\right) \right]$ with derivative $\mathcal {L}_{U_{1}}^{'}(c)$.

We continue to illustrate how effect heterogeneity can affect the integrated hazard difference when the causal effect is time-invariant for each individual. To do so, we let the additive hazard effect modifier $U_{1}$ equal $\mu _{1}$ (${\le }0$, for individuals that benefit) with probability $p_{1}$, $\mu _{2}$ (${\ge }0$, for individuals that are harmed) with probability $p_{2}$ or 0 (for individuals that are not affected). We denote this distribution as the Benefit-Harm-Neutral, $\text {BHN}(p_{1},\mu _{1},p_{2},\mu _{2})$, distribution. Note that it is necessary that $\forall t{:}~ \mathbb {P}(f_{0}(t, U_{0}){<}|\mu _{1})|) = 0$ to guarantee that the hazard rate is always positive for each individual. By Theorem 1 and Lemma 2 with $f_{1}(t,1) = 1$, the SMCHD is equal to $\mathbb {E}\left[ U_{1} \mid T^{1}{\ge }t \right] = \frac{\mu _1 p_1 \exp \left( -t \mu _1\right) +\mu _2 p_2 \exp \left( -t \mu _2\right) }{p_1 \exp \left( -t \mu _1\right) +p_2 \exp \left( -t \mu _2\right) +\left( 1-p_1-p_2\right) },$ and deviates from the constant CHD equal to $\mathbb {E}[U_{1}] = p_{1}\mu _{1} + p_{2}\mu _{2}$. For an RCT, the OHD equals the SMCHD, so that the integrated OHD equals

$$\begin{aligned} B(t) = \int _{0}^{t} \mathbb {E}\left[ U_{1} \mid T^{1}{\ge } s\right] ds = -\log \left( p_{1}(\exp \left( -t\mu _{1}\right) -1)+p_{2}(\exp \left( -t\mu _{2}\right) -1)+1\right) . \end{aligned}$$

**Fig. 1:**
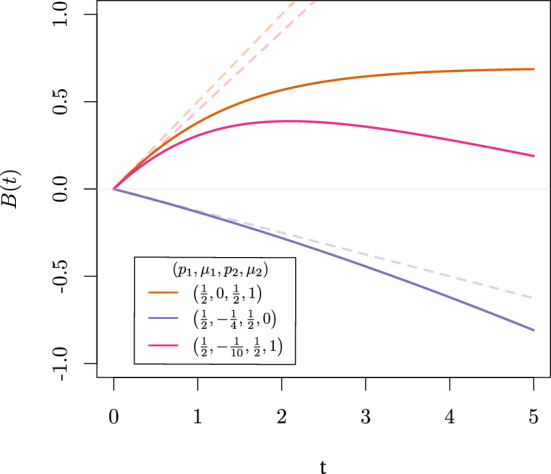
$\int _{0}^{t} \mathbb {E}\left[ U_{1} \mid T^{1}{\ge } s\right] ds$, when $U_{1}~{\sim }~\text {BHN}(p_{1}, \mu _{1}, p_{2}, \mu _{2})$ (solid), and $g(t) = t \mathbb {E}[U_{1}]$ (dashed)

Thus, although the CHD is time-invariant, due to the selection (of $U_{1}$) effect over time *B*(*t*) will not be linear and deviates from the function $g(t) = t \mathbb {E}[U_{1}]$. Three types of curves could be observed as shown in Fig. 1 where for illustration, $p_{1} = p_{2} = 0.5$, so there exist only two levels for the modifier $U_{1}$.

First of all, let’s consider the case where the exposure harms some individuals (for which $U_{1i} = 1$) while others do not respond to the exposure at all ($U_{1i} = 0$); see the orange line in Fig. 1. Initially, *B*(*t*) evolves as $t\mathbb {E}[U_{1}] = 0.5 t$. However, the individuals harmed by the exposure are less likely to survive over time, so the curve’s derivative decreases. In the end, only individuals with $U_{1i} = 0$ are expected to survive so that *B*(*t*) remains constant. Concluding that the exposure initially harms but loses effect over time is false for this case as the effect is time-invariant for each individual.

Secondly, when some individuals do benefit $(U_{1i}~{=-}0.25)$ while others are not affected ($U_{1i} = 0$), the derivative of *B*(*t*) evolves from $-0.125$ to $-0.25$ at the moment only those that benefit are expected to survive, as illustrated with the purple line in Fig. 1. The effect for an individual is again constant and does not become more beneficial over time.

Finally, different individuals in the population might have opposite effects ($U_{1i} = 1$ or $U_{1i}~{=-}0.1$), as illustrated with the pink line in Fig. 1. Initially, the integrated hazard differences increase as the expected effect is harmful. However, over time those individuals with $U_{1i}~{=-}0.1$ are more likely to survive so that $\mathbb {E}[U_{1} \mid T^{1}{\ge } 1]$ changes sign. Finally, only those that benefit are expected to survive, and the curve decreases with a derivative equal to $-0.1$. For this example, it would be false to conclude that the exposure first harms but becomes beneficial over time.

Similar patterns can be observed for a continuous $U_{1}$ distribution, in which case the $\mathbb {E}\left[ U_{1} \mid T^{1}{\ge }t\right]$ will keep decreasing as for example presented in Appendix B.

In summary, if $U_{0} {\mathrel{\perp\!\!\!\perp}} U_{1}$, then the SMCHD will be less or equal to the CHD due to the selection of $U_{1}$. Therefore, decreasing or constant *B*(*t*) curves that at some point increase again can not be explained by the selection of $U_{1}$ since individuals with less beneficial values of $U_{1}$ are expected to survive shorter. However, if , such a pattern of the *B*(*t*) curve can still occur when the CHD is time-invariant as we will show next.

### Dependent $U_{0}$ and $U_{1}$

**Fig. 2:**
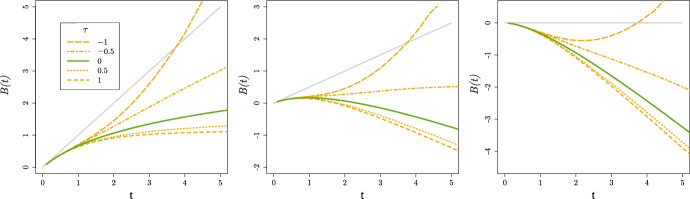
Integrated hazard difference, *B*(*t*), when $f_{0}(t,U_{0i}) = \ell + U_{0i}t^{2}$, $U_{0i}~{\sim }~\Gamma (1,1)$, $(U_{1i}+ \ell ) ~{\sim }~\Gamma (1,1)$ for $\ell$ equal to 0 (left), $\tfrac{1}{2}$ (middle) and 1 (right) and different Kendall’s $\tau$ for $U_{0i}$ and $U_{1i}$. The lines for $\tau = 0$ were already presented in Fig. 5 in Appendix B. Furthermore, $g(t) = t \mathbb {E}[U_{1}]$ are presented as gray lines

The bivariate joint distribution function of $U_{0}$ and $U_{1}$, $F_{(U_{0}, U_{1})}$, can be written using the marginal distribution functions and a copula *C* (Sklar 1959). As such,

$$\begin{aligned} F_{(U_{0},U_{1})}(u_{0},u_{1}) = C\left( F_{U_{0}}(u_{0}), F_{U_{1}}(u_{1})\right) \end{aligned}$$

and the Kendall’s $\tau$ correlation coefficient of $U_{0}$ and $U_{1}$ can be written as a function of the copula (Nelsen 2006). For the next example, we consider cause-effect relations for which SCM (2) applies with

8 $$\begin{aligned} f_{0}(t,U_{0i}) = \ell + U_{0i}t^{2},\end{aligned}$$

and again

9 $$\begin{aligned} f_{1}(t,U_{1i},a)=U_{1i}a,\end{aligned}$$

while $U_{0i} ~{\sim }~\Gamma (1,1)$ and $(U_{1i}+\ell ) ~{\sim }~\Gamma (1,1)$, so that the hazard is nonnegative for each individual. To illustrate how the dependence can affect the integrated SMCHD for the settings presented in Fig. 5 in Appendix B, we use a Gaussian copula

$$\begin{aligned} C(x,y) = \Phi _{2,\rho }(\Phi ^{-1}(x), \Phi ^{-1}(y)), \end{aligned}$$

where $\Phi$ and $\Phi _{2,\rho }$ are the standard normal and standard bivariate normal with correlation $\rho$ cumulative distribution functions, respectively. In Fig. 2, for $\rho \in \{ -1, \sin ( -0.25\pi) , 0, \sin ( 0.25\pi ) , 1\}$ (such that $\tau \in \{ -1, -0.5, 0, 0.5, 1\}$) and $\ell \in \{0, 0.5, 1 \}$, we present the integrated SMCHD at time *t* that equals $\int _{0}^{t} \left( \mathbb {E}[ U_{1} \mid T^{1}{\ge } s] + \mathbb {E}[ U_{0}s^{2} \mid T^{1}{\ge } s] -\mathbb {E}[U_{0}s^{2} \mid T^{0}{\ge } s] \right) ds$ by Theorem 1. The conditional expectations are derived empirically from simulations $(n{=}10{,}000)$, and the integral is approximated by taking discrete steps of size 0.1. For completeness, the survival curves of the potential outcomes can be found in Fig. 6 in Appendix C.

The difference between the integrated OHD and SMCHD increases when $\tau >0$ (compared to $\tau = 0$). On the other hand, for $\tau <0$, the difference is smaller most of the time than for $\tau = 0$ since favourable $U_{1}$ are expected to occur with unfavourable levels of $U_{0}$. Moreover, for $\tau = -1$, at larger *t*, we observe that the difference can even change sign. For $\tau \ne 0$, the SMCHD might thus be larger than the CHD, so the SMCHD is not a theoretical lower bound for the CHD. Note that if , the integrated SMCHD depends on the functional form of $f_{0}$. In Fig. 7 in Appendix C, the results for $f_{0}(t, U_{0i}) = \ell + U_{0i}\frac{t^{2}}{20}$ are presented where the effect of the dependence is limited and the corresponding survival curves of the potential outcomes are presented in Fig. 8.

## Case study: the Radiation Therapy Oncology Group trial

**Fig. 3:**
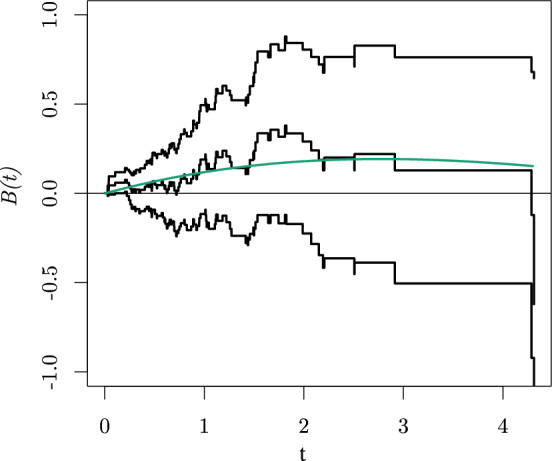
Estimated *B*(*t*) and corresponding $95\%$ confidence interval (black). Furthermore, the expected evolution of *B*(*t*) when $\lambda _{i}^{a}(t) = f_{0}(t,U_{0i})+U_{1i}a$ and $U_{1i} ~{\sim }~\text {BHN}(0.5, -0.1, 0.5, 0.4)$ is presented (green)

With the findings of the previous section we will reflect on a data analysis of an actual case study to illustrate why it is important for a practioner to be aware of the possible difference between the SMCHD and CHD. We consider a large clinical trial carried out by the Radiation Therapy Oncology Group as described by Kalbfleisch and Prentice (2002, Section 1.1.2 and Appendix A) and also presented by Aalen (1989). From the patients with squamous cell carcinoma (a form of skin cancer) of 15 sites in the mouth and throat from 16 participating institutions, our focus is only on two sites (faucial arch and pharyngeal tongue) and patients from the six largest institutions. All participants were randomly assigned to radiation therapy alone or combined with a chemotherapeutic agent. So, we are interested in the causal effect of the chemotherapeutic agent in addition to radiation therapy on survival. If the causal mechanism can be parameterized with SCM (2) without effect heterogeneity, i.e. 

$$\begin{aligned} \lambda _{i}^{a}(t) = f_{0}(t,U_{0i})+f_{1}(t,a), \end{aligned}$$

and the randomization was properly executed, implying $N_{Ai} {\mathrel{\perp\!\!\!\perp}} U_{0i}$, then, by Theorem 1, the OHD equals the CHD. Moreover, the CHD can be unbiasedly estimated by fitting Aalen’s additive hazard model. We did so by using the aalen() function from the package timereg in R. The estimated cumulative regression function (and a corresponding $95\%$ confidence interval) of treatment combined with a chemotherapeutic agent is presented by the black lines in Fig. 3.

**Fig. 4:**
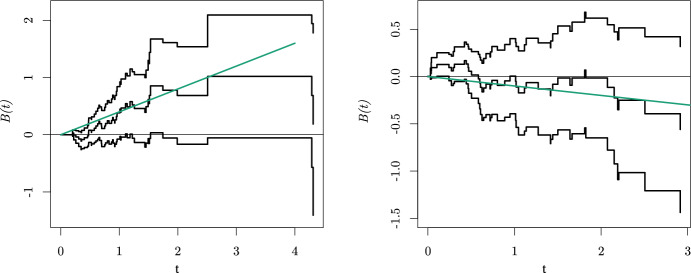
Estimated *B*(*t*) and corresponding $95\%$ confidence interval (black) for patients with tumours located at the faucial arch (left) and pharyngeal tongue (right), respectively. Furthermore, the *B*(*t*) for a homogeneous population is presented (green) equal to 0.4*t* (left) and $-0.1t$ (right) for comparison

**Fig. 5:**
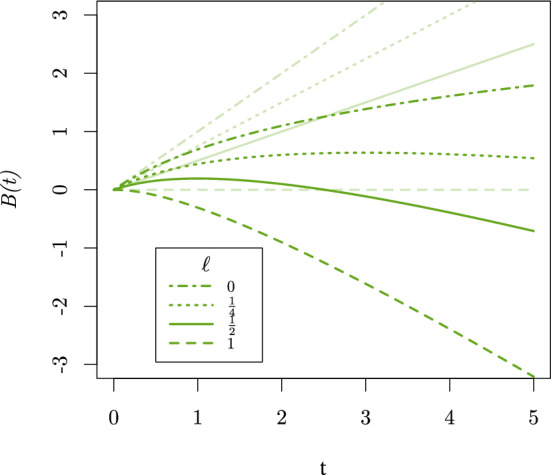
$\int _{0}^{t} \mathbb {E}\left[ U_{1} \mid T^{1}{\ge } s\right] ds$ (thick lines), when $U_{1}+\ell ~{\sim }~\Gamma (1,1)$ and $g(t) = t \mathbb {E}[U_{1}]$ (transparent lines)

**Fig. 6:**
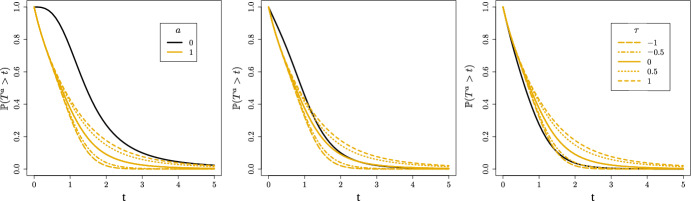
Survival curves for $Y^{1}$ and $Y^{0}$ when $f_{0}(t,U_{0i}) = \ell + U_{0i}t^{2}$, $U_{0i}~{\sim }~\Gamma (1,1)$, $(U_{1i}+ \ell ) ~{\sim }~\Gamma (1,1)$ for $\ell$ equal to 0 (left), $\tfrac{1}{2}$ (middle) and 1 (right) and different Kendall’s $\tau$ for $U_{0i}$ and $U_{1i}$

**Fig. 7:**
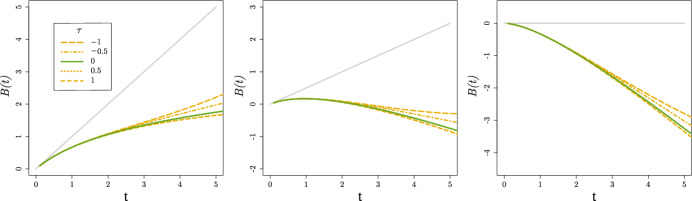
*B*(*t*), when $f_{0}(t,U_{0i}) = \ell + U_{0i}\tfrac{t^{2}}{20}$, $U_{0i}~{\sim }~\Gamma (1,1)$, $(U_{1i}+ \ell ) ~{\sim }~\Gamma (1,1)$ for $\ell$ equal to 0 (left), $\tfrac{1}{2}$ (middle) and 1 (right) and different Kendall’s $\tau$ for $U_{0i}$ and $U_{1i}$. The lines for $\tau = 0.5$ and $\tau = 1$ do overlap, and the lines for $\tau = 0$ were already presented in Fig. 5. Furthermore, $g(t) = t \mathbb {E}[U_{1}]$ are presented as gray lines

**Fig. 8:**
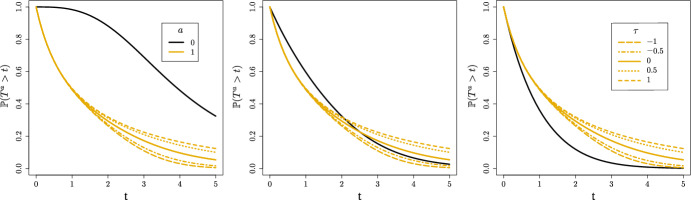
Survival curves for $Y^{1}$ and $Y^{0}$ when $f_{0}(t,U_{0i}) = \ell + U_{0i}\tfrac{t^{2}}{20}$, $U_{0i}~{\sim }~\Gamma (1,1)$, $(U_{1i}+ \ell ) ~{\sim }~\Gamma (1,1)$ for $\ell$ equal to 0 (left), $\tfrac{1}{2}$ (middle) and 1 (right) and different Kendall’s $\tau$ for $U_{0i}$ and $U_{1i}$

In the absence of effect heterogeneity (ignoring the statistical uncertainty), one could now conclude that initially adding the chemotherapy is expected to harm a patient as *B*(*t*) takes on positive values and that the exposure loses its harmful effect over time as the derivative of *B*(*t*) decreases over time. Following a similar reasoning, Aalen et al. (2008, pp. 160-161) discuss a conclusion on the effect of N-stage (an index of lymph node metastasis) on survival that may be drawn by practitioners based on the same dataset (while also including patients with a tumour located at the tonsillar fossa): *“The regression plot shows that this* [non-significant P-value for a zero-effect of N-stage from a Cox analysis] *is due to a strong initial positive effect being “watered down" by a lack of, or even a slightly negative effect after one year. Hence, not taking into consideration the change in effect over time may lead to missing significant effects."*. However, if in reality

$$\begin{aligned} \lambda _{i}^{a}(t) = f_{0}(t,U_{0i})+f_{1}(t,U_{1i},a), \end{aligned}$$

where $f_{1}(t,U_{1i},0)=0$, the observed time-varying effect can also result from the modifier $U_{1i}$ selection. For example, when $\lambda _{i}^{a}(t) = f_{0}(t, U_{0i})+U_{1i}a$, and $U_{1i} ~{\sim }~\text {BHN}(0.5, -0.1, 0.5, 0.4)$, by Theorem 1, this pattern is expected (see the green line in Fig. 3) while the actual causal effect is time-invariant for each individual. The CHD equals 0.15 at each time point, but over time individuals that are harmed by the chemotherapy ($U_{1i} = 0.4$) are less likely to survive so that the SMCHD converges towards $-0.1$ (the effect for individuals that benefit from the chemotherapy). When we perform a stratified analysis by site in the oropharynx (where randomization remains), we observe that the effect of chemotherapy might have opposite effects for tumours located in the faucial arch and on the pharyngeal tongue, see Fig. 4. The tumour location could thus be the individual modifier underlying the BHN distribution. For this case study, we cannot be sure whether the effect of chemotherapy depends on the tumour location due to statistical uncertainty. However, it became clear that when statistical uncertainty is not the issue, it will be impossible to distinguish between a time-varying causal effect and a selection effect (of an unmeasured modifier) from data. Both phenomena can give rise to the same *B*(*t*).

## Discussion

The additive hazard model gives better interpretable estimates of causal effects than the proportional hazard model (Aalen et al. 2015). As discussed by Aalen et al. (2015), the model assumes that the additive part of the hazard involving the exposure (or treatment) is not affected by any other individual feature. Otherwise, if such effect heterogeneity at the hazard scale exists, we have shown that the SMCHD deviates from the CHD of interest. For an RCT, and independent censoring, a time-varying observed hazard difference can be the result of either an actual time-varying causal effect or of the selection of favourable effect-modifier levels over time. Therefore, it is impossible to distinguish these scenarios based on data without making untestable assumptions. It is important to remark that for cause-effect relations that can be parameterized with SCM (2) where $U_{1}$ is degenerate (in which case the OHD equals the CHD), contrary to the individual hazard differences, the difference of the potential survival times, $T^{1}-T^{0}$ can be heterogeneous. So, heterogeneous effects can still exist under Aalen’s additive hazard model.

In the presented examples and the case study, we have illustrated that one should be very careful when concluding that the effect decreases over time based on the cumulative regression function, as this might result from the selection. The size of the bias depends on how much the distribution $F_{U_{1} \mid T^{1}{\ge }t}$ changes over time. If the $U_{1}$ is low in variability, the bias will be small. When analyzing data from an RCT with an additive hazard model, it can thus be helpful to adjust for potential effect modifiers to reduce the remaining variability of unmeasured effect modifiers. We want to remark that for cause-effect relations that cannot be described by SCM (2), the CHD is not the appropriate estimand to quantify the causal effect, which is then a more serious concern than that the complicated causal interpretation of the observed hazard difference.

Even in the absence of confounding, the hazard difference and the hazard ratio (as discussed in Post et al. (2024)) have a difficult causal interpretation. Instead, contrasts of the survival probabilities, the median, or the restricted mean survival time, have clear causal interpretations and should thus be used to quantify the causal effect on time-to-event outcomes as suggested by others (Hernán 2010; Stensrud et al. 2018; Bartlett et al. 2020; Young et al. 2020). Nevertheless, (additive) hazard models can still be used for causal inference to derive these appropriate estimands (Ryalen et al. 2018).

## Appendix A: Proofs

### Appendix A.1: Proof of Theorem 1

#### Proof

By causal consistency (Hernán and Robins 2020),

$$\begin{aligned} \lim _{h\rightarrow 0}h^{-1}\mathbb {P}\left( T ~{\in }~[t,t+h) \mid T{\ge }t, A{=}a \right) = \lim _{h\rightarrow 0}h^{-1}\mathbb {P}\left( T^{a} ~{\in }~[t,t+h) \mid T^{a}{\ge }t, A{=}a \right) \end{aligned}$$

As $N_{A} {\mathrel{\perp\!\!\!\perp}} U_{0}, U_{1}, N_{T}$, there is no confounding and $T^{a} {\mathrel{\perp\!\!\!\perp}} A$, so that

$$\begin{aligned} \lim _{h\rightarrow 0}h^{-1}\mathbb {P}\left( T ~{\in }~[t,t+h) \mid T{\ge }t, A{=}a \right) = \lim _{h\rightarrow 0}h^{-1}\mathbb {P}\left( T^{a} ~{\in }~[t,t+h) \mid T^{a}{\ge }t, \right) . \end{aligned}$$

By the law of total probability,

$$\begin{aligned}{} & {} \lim _{h\rightarrow 0}h^{-1}\mathbb {P}\left( T^{a} ~{\in }~[t,t+h) \mid T^{a}{\ge }t \right) \\ {}{} & {} \quad = \lim _{h\rightarrow 0}\int h^{-1}\mathbb {P}\left( T^{a} ~{\in }~[t,t+h) \mid T^{a}{\ge }t, U_{0}, U_{1} \right) dF_{U_{0}, U_{1} \mid T^{a}{\ge }t} \end{aligned}$$

First, we focus on the integrand,

$$\begin{aligned}&h^{-1}\mathbb {P}\left( T^{a} ~{\in }~[t,t+h) \mid T^{a}{\ge }t, U_{0}, U_{1} \right) \\&= h^{-1}\frac{\mathbb {P}\left( T^{a}{\ge } t \mid U_{0}, U_{1} \right) - \mathbb {P}\left( T^{a}{\ge } t + h \mid U_{0}, U_{1} \right) }{\mathbb {P}\left( T^{a}{\ge } t \mid U_{0}, U_{1} \right) } \\ {}&= h^{-1}\left( 1-\frac{\mathbb {P}\left( T^{a}{\ge } t + h \mid U_{0}, U_{1} \right) }{\mathbb {P}\left( T^{a}{\ge } t \mid U_{0}, U_{1} \right) }\right) \\ {}&= h^{-1}\left( 1-\frac{\exp \left( - \int _{0}^{t+h} f_{0}(s,U_{0})+f_{1}(s,U_{1},a) ds \right) }{\exp \left( - \int _{0}^{t} f_{0}(s,U_{0})+f_{1}(s,U_{1},a) ds \right) }\right) \\ {}&= h^{-1}\left( 1-\exp \left( - \int _{t}^{t+h} f_{0}(s,U_{0})+f_{1}(s,U_{1},a) ds \right) \right) \end{aligned}$$

For monotonic conditional hazard functions, if $h_{2}<h_{1}$, then

$$\begin{aligned}&h_{1}^{-1}\left( 1- \exp \left( - \int _{t}^{t+h_{1}} f_{0}(s,U_{0})+f_{1}(s,U_{1},a) ds \right) \right) \\ {}&\quad \le h_{2}^{-1}\left( 1- \exp \left( - \int _{t}^{t+h_{2}} f_{0}(s,U_{0})+f_{1}(s,U_{1},a) ds \right) \right) \end{aligned}$$

or

$$\begin{aligned}&h_{1}^{-1}\left( 1- \exp \left( - \int _{t}^{t+h_{1}} f_{0}(s,U_{0})+f_{1}(s,U_{1},a) ds \right) \right) \\ {}&\quad \ge h_{2}^{-1}\left( 1- \exp \left( - \int _{t}^{t+h_{2}} f_{0}(s,U_{0})+f_{1}(s,U_{1},a) ds \right) \right) \end{aligned}$$

as the average integrated conditional hazard over the interval increases (or decreases). Moreover,

$$\begin{aligned} \lim _{h\rightarrow 0} h^{-1}\mathbb {P}\left( T^{a} \in [t,t+h) \mid T^{a}{\ge } t, U_{0}, U_{1} \right) = f_{0}(s,U_{0}) + f_{1}(s,U_{1},a)\ge 0. \end{aligned}$$

Then, the limit and integral can be interchanged by directly applying the monotone convergence theorem.

For non-monotone conditional hazard functions, when Condition 1 applies, for every *t*, there exist a $\tilde{h}$ so that $\forall h^{*} \in (0,\tilde{{}h}){:}~ \mathbb {E}\left[ f_{0}(t+h^{*},U_{0}) + f_{1}(t+h^{*},U_{1},a) \mid T^{a}{\ge } t \right] < \infty$. Moreover, let $t^{*}{=}~ \mathop {\mathrm {arg\,max}}\limits _{s \in (t, t + \tilde{{}h})} f_{0}(s,U_{0}) + f_{1}(s,U_{1},a),$ so that for $h<\tilde{h}{:}~$

$$\begin{aligned}h^{-1}\mathbb {P}\left( T^{a} ~{\in }~[t,t+h) \mid T^{a}{\ge }t, U_{0}, U_{1} \right) \le h^{-1}\left( 1- \exp \left( - h\left( f_{0}(t^{*},U_{0})+f_{1}(t^{*},U_{1},a)\right) \right) \right) . \end{aligned}$$

Using the power series definition of the exponential function,

$$\begin{aligned}&h^{-1}\mathbb {P}\left( T^{a} ~{\in }~[t,t+h) \mid T^{a}{\ge }t, U_{0}, U_{1} \right) \\&\le h^{-1}\left( 1- \frac{1}{\sum _{k=0}^{\infty }h^{k} (f_{0}(t^{*},U_{0})+f_{1}(t^{*},U_{1},a))^{k}\tfrac{1}{k!}}\right) \\&= h^{-1} \frac{\sum _{k=1}^{\infty }h^{k} (f_{0}(t^{*},U_{0})+f_{1}(t^{*},U_{1},a))^{k}\tfrac{1}{k!}}{\sum _{k=0}^{\infty }h^{k} (f_{0}(t^{*},U_{0})+f_{1}(t^{*},U_{1},a))^{k}\tfrac{1}{k!}}\\ {}&= \left( f_{0}(t^{*},U_{0})+f_{1}(t^{*},U_{1},a)\right) \frac{\sum _{k=1}^{\infty }h^{k-1} (f_{0}(t^{*},U_{0})+f_{1}(t^{*},U_{1},a))^{k-1}\tfrac{1}{k!}}{\sum _{k=0}^{\infty }h^{k} (f_{0}(t^{*},U_{0})+f_{1}(t^{*},U_{1},a))^{k}\tfrac{1}{k!}}\\ {}&= \left( f_{0}(t^{*},U_{0})+f_{1}(t^{*},U_{1},a)\right) \frac{\sum _{k=0}^{\infty }h^{k} (f_{0}(t^{*},U_{0})+f_{1}(t^{*},U_{1},a))^{k}\tfrac{1}{(k+1)!}}{\sum _{k=0}^{\infty }h^{k} (f_{0}(t^{*},U_{0})+f_{1}(t^{*},U_{1},a))^{k}\tfrac{1}{k!}}\\ {}&< f_{0}(t^{*},U_{0})+f_{1}(t^{*},U_{1},a). \end{aligned}$$

Moreover, $\mathbb {E}\left[ f_{0}(t^{*},U_{0}) + f_{1}(t^{*},U_{1},a) \mid T^{a}{\ge }t \right] < \infty$ when $\mathbb {E}[ f_{0}(t+h,U_{0}) + f_{1}(t+h, U_{1},a) \mid T^{a}{\ge }t ] < \infty$ for all $h ~{\in }~(0,\tilde{{}h})$. By application of the dominated convergence theorem, we can change the order of the limit and integral and conclude,

$$\begin{aligned} \lim _{h\rightarrow 0}h^{-1}\mathbb {P}\left( T^{a} ~{\in }~[t,t+h) \mid T^{a}{\ge }t \right) = \mathbb {E}\left[ f_{0}(t,U_{0})+f_{1}(t,U_{1},a) \mid T^{a}{\ge }t\right] . \end{aligned}$$

If $U_{0} {\mathrel{\perp\!\!\!\perp}} U_{1}$, by Lemma 1,

$$\begin{aligned} \mathbb {E}[f_{0}(t,U_{0}) + f_{1}(t,U_{1},1) \mid T^{1}{\ge }t]-\mathbb {E}[f_{0}(t,U_{0}) \mid T^{0}{\ge }t] = \mathbb {E}\left[ f_{1}(t,U_{1},1) \mid T^{1}{\ge }t\right] , \end{aligned}$$

and

$$\begin{aligned}&\lim _{h\rightarrow 0}h^{-1}\mathbb {P}\left( T ~{\in }~[t,t+h) \mid T{\ge }t, A{=}1 \right) -\lim _{h\rightarrow 0}h^{-1}\mathbb {P}\left( T~{\in }~[t,t+h) \mid T{\ge }t, A{=}0 \right) \\ {}&= \mathbb {E}\left[ f_{1}(t,U_{1},1) \mid T^{1}{\ge }t\right] . \end{aligned}$$

$$\square$$

### Appendix A.2: Proof of Lemma 1

#### Proof

$$\begin{aligned}&\mathbb {E}\left[ f_{0}(t,U_{0}) \mid T^{a}{\ge }t\right] \\ {}&= \int f_{0}(t,u_{0}) dF_{U_{0} \mid T^{a}{\ge }t}(u_{0})\\ {}&= \int f_{0}(t,U_{0}) \frac{\mathbb {P}(T^a{\ge }t \mid U_{0}{=}u_{0})}{\mathbb {P}(T^{a}{\ge } t)}dF_{U_{0}}(u_{0})\\ {}&= \int f_{0}(t,U_{0}) \frac{\int \exp \left( -(\int _{0}^{t}f_{0}(s,U_{0})ds+\int _{0}^{t}f_{1}(s,U_{1},a)ds )\right) dF_{U_{1}|U_{0}{=}u_{0}}(u_{1})}{\int \int \exp \left( -(\int _{0}^{t}f_{0}(k_{0},s)ds + \int _{0}^{t}f_{1}(k_{1},a,s)ds)\right) dF_{U_{1} \mid U_{0}{=}k_{0}}(k_{1}) dF_{U_{0}}(k_{0})} dF_{U_{0}}(u_{0})\\ {}&= \int f_{0}(t,U_{0}) \frac{\exp \left( -\int _{0}^{t}f_{0}(s,U_{0})ds\right) \left( \int \exp \left( -\int _{0}^{t}f_{1}(s,U_{1},a)ds \right) dF_{U_{1} \mid U_{0}{=}u_{0}}(u_{1})\right) }{\int \exp \left( -\int _{0}^{t}f_{0}(k_{0},s)ds \right) \left( \int \exp \left( - \int _{0}^{t}f_{1}(k_{1},a,s)ds\right) dF_{U_{1} \mid U_{0}{=}k_{0}}(k_{1})\right) dF_{U_{0}}(k_{0})} dF_{U_{0}}(u_{0}). \end{aligned}$$

Moreover, if $U_{0} {\mathrel{\perp\!\!\!\perp}} U_{1}$, then

$$\begin{aligned}&\mathbb {E}\left[ f_{0}(t,U_{0}) \mid T^{a}{\ge }t\right] \\ {}&= \int f_{0}(t,U_{0}) \frac{\exp \left( -\int _{0}^{t}f_{0}(s,U_{0})ds\right) \left( \int \exp \left( -\int _{0}^{t}f_{1}(s,U_{1},a)ds \right) dF_{U_{1}}(u_{1})\right) }{\int \exp \left( -\int _{0}^{t}f_{0}(k_{0},s)ds \right) \left( \int \exp \left( - \int _{0}^{t}f_{1}(k_{1},a,s)ds\right) dF_{U_{1} }(k_{1})\right) dF_{U_{0}}(k_{0})} dF_{U_{0}}(u_{0})\\ {}&= \int f_{0}(t,U_{0}) \frac{\exp \left( -\int _{0}^{t}f_{0}(s,U_{0})ds\right) \left( \int \exp \left( -\int _{0}^{t}f_{1}(s,U_{1},a)ds \right) dF_{U_{1}}(u_{1})\right) }{\left( \int \exp \left( -\int _{0}^{t}f_{0}(k_{0},s)ds\right) dF_{U_{0}}(k_{0}) \right) \left( \int \exp \left( - \int _{0}^{t}f_{1}(k_{1},a,s)ds\right) dF_{U_{1} }(k_{1})\right) } dF_{U_{0}}(u_{0})\\ {}&= \int f_{0}(t,U_{0}) \frac{\exp \left( -\int _{0}^{t}f_{0}(s,U_{0})ds\right) }{\int \exp \left( -\int _{0}^{t}f_{0}(k_{0},s)ds\right) dF_{U_{0}}(k_{0})} dF_{U_{0}}(u_{0}) \\ {}&= \mathbb {E}\left[ f_{0}(t,U_{0}) \mid T^{0}{\ge }t\right] . \end{aligned}$$

$$\square$$

### Appendix A.3: Proof of Lemma 2

#### Proof

If $U_{0} {\mathrel{\perp\!\!\!\perp}} U_{1}$, by Bayes rule, the probability density of $U_{1}$ given $T^{1}\ge t$, $f(u_{1} {\mid } T^{1}{\ge }t)$, equals

$$\begin{aligned}&f(u_{1} {\mid } T^{1}{\ge }t)\\ {}&= \frac{\mathbb {P}(T^{1}{\ge } t \mid U_{1}{=}u_{1})f(u_{1})}{\int \mathbb {P}(T^{1}{\ge } t \mid U_{1}=u_{1})dF_{U_{1}}(u_{1})}\\ {}&= \frac{\int \exp \left( -\left( \int _{0}^{t}f_{0}(s,U_{0})+u_{1}f_{1}(a,s) ds\right) \right) dF_{U_{0}}(u_{0})f(u_{1})}{\int \int \exp \left( -\left( \int _{0}^{t}f_{0}(k_{0},s)+k_{1}f_{1}(a,s)ds\right) \right) dF_{U_{1}}(k_{1})dF_{U_{0}}(k_{0})}\\&= \frac{\exp \left( -\int _{0}^{t}u_{1}f_{1}(a,s) ds\right) \int \exp \left( -\int _{0}^{t}f_{0}(s,U_{0}) ds\right) dF_{U_{0}}(u_{0})f(u_{1})}{\int \exp \left( -k_{1}f_{1}(a,s)ds\right) dF_{U_{1}}(k_{1})\int \exp \left( -\int _{0}^{t}f_{0}(k_{0},s)ds\right) dF_{U_{0}}(k_{0})}\\ {}&= \frac{\exp \left( -\int _{0}^{t}u_{1}f_{1}(a,s) ds\right) f(u_{1})}{\int \exp \left( -k_{1}f_{1}(a,s)ds\right) dF_{U_{1}}(k_{1})}. \end{aligned}$$

So that the Laplace transform of $f(u_{1} {\mid } T^{1}{\ge }t)$ can be written as

$$\begin{aligned} \mathcal {L}_{U_{1}{\mid }T^{1}{\ge }t}(c)&= \mathbb {E}[\exp \left( -U_{1}c\right) \mid T^{1}{\ge }t]\\ {}&= \int \exp \left( -u_{1}c\right) dF_{U_{1} \mid T^{1}{\ge }t}(u_{1})\\ {}&= \int \exp \left( -u_{1}c\right) \frac{\exp \left( -\int _{0}^{t}u_{1}f_{1}(s,1) ds\right) }{\int \exp \left( -k_{1}f_{1}(s,1)ds\right) dF_{U_{1}}(k_{1})}dF_{U_{1}}(u_{1})\\&= \frac{\int \exp \left( -u_{1}(c+\int _{0}^{t}f_{1}(s,1) ds)\right) }{\int \exp \left( -k_{1}f_{1}(s,1)ds\right) dF_{U_{1}}(k_{1})}dF_{U_{1}}(u_{1})\\&= \frac{\mathbb {E}\left[ \exp \left( -U_{1}(c+\int _{0}^{t}f_{1}(s,1) ds)\right) \right] }{\mathbb {E}\left[ \exp \left( -U_{1}f_{1}(s,1)ds\right) \right] }\\&= \frac{\mathcal {L}_{U_{1}}(c+\int _{0}^{t}f_{1}(s,1) ds)}{\mathcal {L}_{U_{1}}(\int _{0}^{t}f_{1}(s,1) ds)}. \end{aligned}$$

Since for a random variable *X*, $\mathbb {E}[X] = -\mathcal {L}_{X}^{'}(0)$,

$$\begin{aligned} \mathbb {E}[U_{1} \mid T^{1}{\ge }t] = -\mathcal {L}_{U_{1} \mid T^{1}{\ge }t}^{'}(0) = -\frac{\mathcal {L}_{U_{1}}^{'}(\int _{0}^{t}f_{1}(s,1)ds)}{\mathcal {L}_{U_{1}}(\int _{0}^{t}f_{1}(s,1)ds)}. \end{aligned}$$

$$\square$$

## Appendix B: Continuous $U_{1}$ distribution ($U_{0} {\mathrel{\perp\!\!\!\perp}} U_{1}$)

Let us consider cause-effect relations for which SCM (2) applies with

10 $$\begin{aligned} f_{1}(t,U_{1i},a)=U_{1i}a, \end{aligned}$$

and $U_{0} {\mathrel{\perp\!\!\!\perp}} U_{1}$. Moreover, let $(U_{1}+\ell ) ~{\sim }~\Gamma (k, \theta )$, then $\mathcal {L}_{U_{1}+\ell }(c) = (1+\theta c)^{-k}$. By lemma 2,

$$\begin{aligned} \mathbb {E}\left[ U_{1} \mid T^{1}{\ge }t\right] = \frac{\theta k}{\theta t +1} - \ell , \end{aligned}$$

and

$$\begin{aligned} B(t) = k\log (\theta t + 1) - \ell t. \end{aligned}$$

The *B*(*t*) are presented over time in Fig. 5 for $\theta = k = 1$ and $\ell ~{\in }~\{0, \tfrac{1}{4}, \tfrac{1}{2}, 1 \}$.

## Appendix C: Figures dependent $U_{0}$ and $U_{1}$

The additional figures for $f_{0}(t,U_{0i}) = \ell + U_{0i}\frac{t^{2}}{20}$.
